# Effect of depression and serotonin reuptake inhibitors antidepressant treatment during pregnancy on protein expression in the human placenta: A quantitative proteomics analysis

**DOI:** 10.1371/journal.pone.0322090

**Published:** 2025-12-11

**Authors:** Linda Ok, Leanne Ohlund, Amy M. Inkster, Kayleigh S. J. Campbell, Maria S. Peñaherrera, Ursula Brain, Lekha Sleno, Wendy P. Robinson, Amadou Barry, Tim F. Oberlander, Cathy Vaillancourt

**Affiliations:** 1 Institut national de la recherche scientifique (INRS) - Centre Armand-Frappier Santé Biotechnologie, Laval, Quebec, Canada; 2 Research Centre and CIUSSS du Nord-de-l’Île-de-Montréal, Montréal, Quebec, Canada; 3 Chemistry Department/CERMO-FC, Université du Québec à Montréal, Montréal, Quebec, Canada; 4 Department of Medical Genetics, University of British Columbia, Vancouver, British Columbia, Canada; 5 BC Children’s Hospital Research Institute, Vancouver, British Columbia, Canada; 6 Department of Pediatrics, University of British Columbia, Vancouver, British Columbia, Canada; 7 Unité mixte de recherche INRS-UQAC en santé durable, Chicoutimi, Quebec, Canada; 8 School of Population and Public Health, University of British Columbia, Vancouver, British Columbia, Canada; King Saud University / Zagazig University, EGYPT

## Abstract

Depression is one of the most prevalent mental health disorders affecting pregnant individuals, and serotonin reuptake inhibitors (SRIs) are the most prescribed medications to treat depressive symptoms during pregnancy. Both depression and SRI exposure may have developmental impacts, and as randomizing exposures and non-treatment is not feasible in humans, distinguishing the effect of each factor often remains challenging. To date, much of what guides clinical practice stems from reports of pregnant individuals and fetal/infant outcomes, overlooking the significant role of the placenta in maintaining a healthy pregnancy and mother-fetal health. In this study, we explored the effect of depression and SRI antidepressant treatment during pregnancy on the placental proteome. A shotgun proteomics method with liquid chromatography and tandem mass spectrometry (LC-MS/MS) was performed. A cohort of pregnant individuals (n = 82) was recruited in their 2^nd^ trimester, and clinician-rated mood symptoms measured using the Hamilton Depression Rating Scale (HAM-D) were obtained from them during pregnancy to identify three exposure groups: non-depressed; depressed/non-SRI-treated; depressed/SRI-treated. Differential protein expression and over-representation analyses on the placentas of the group with depression/non-SRI compared with placentas from healthy individuals showed an increase in antioxidant enzymes and various senescence-associated secretory phenotypes (SASP) as well as a decrease in histone proteins. Such protein expression patterns are potentially indicative of placental senescence. Our findings reveal that depression not treated with SRIs is associated with the upregulation of proteins involved in platelet activation, degranulation, coagulation cascade, and amyloid fiber formation in the placenta. In contrast, when comparing placentas from the depressed group treated with SRIs to those with depressive symptoms without SRI treatment, we observed a downregulation of proteins related to senescence, amyloid fiber formation, and platelet activation in the SRI-treated group. This study suggests that treatment with SRIs may prevent the placental alterations observed in antenatal depression, such as placental senescence, platelet activation, and amyloid fiber formation, and, ultimately, pregnancy and fetal outcomes. Further studies are needed to confirm these findings.

## 1. Introduction

Antenatal depression is a disorder that affects 15 ~ 20% of pregnant people worldwide [1]. Antenatal depression hinders well-being during pregnancy and is associated with adverse birth outcomes such as low birth weight, intrauterine growth restriction, and increased maternal and fetal morbidity and mortality, and has a long-term effect on a child’s physical, cognitive, and behavioral development [2,3]. Pregnant people with antenatal depression often experience sleep disturbances and poor nutrition, which compromises their health [2,4]. Furthermore, antenatal depression increases the risk of postpartum depression, altered fetal development, and maternal suicide [3,5]. Serotonin reuptake inhibitors (SRIs) are commonly used pharmacological treatments, with 3.0% (95% CI 2.3;3.7) international prevalence of use during pregnancy [6]. While SRI treatment during pregnancy is deemed safe by the regulatory agencies, there are numerous studies suggesting the contrary [7,8]. In-utero exposure to common SRIs such as fluoxetine, sertraline, citalopram, and paroxetine has been associated with cardiovascular and musculoskeletal defects in the fetus [9–11]. Furthermore, increased risk of preterm birth, low birth weight, and developmental disorders has been indicated in neonates exposed to SRI during pregnancy [12,13].

To understand the changes associated with antenatal depression, proteomics approaches have been used on biological samples such as plasma and serum obtained from individuals with depression during pregnancy [14,15]. The placenta is a fetal organ that regulates and supports maternal-fetal gas and nutrient exchange, waste removal, immunity, and hormone production and regulation during pregnancy. Proteomics data derived from placental tissues can provide information on the mother’s and fetus’s molecular and physiological state, including the changes associated with pregnancy-related diseases such as pre-eclampsia and gestational diabetes [16,17]. Compared to the relatively static genome, the proteome is highly variable and complex due to its existence in multiple isoforms and responsiveness to environmental challenges [17]. Thus, we hypothesized that the proteomics analysis of the placental tissue could identify molecular and physiological changes in the placenta in response to the depressive symptoms and the SRI treatment during pregnancy. The effect of antenatal depression and SRIs treatment was assessed by comparing 1) pregnant individuals without depressive symptoms to those with mild to moderately depressive symptoms without SRI treatment and ii) pregnant individuals with mild to moderately depressive symptoms without SRIs to those with mild to moderately depressive symptoms treated with SRIs.

## 2. Materials and methods

### 2.1. Participants and samples stratification

Placentas were collected from an observational study cohort where participants were selected based on their exposure to depression and SRI treatment throughout the pregnancy. In the late second trimester, 83 pregnant people were recruited from the Reproductive Mental Health Clinic at British Columbia Women’s Hospital and Health Center, community midwives, or family physicians in metropolitan Vancouver, Canada (from February 2013–July 2017). The ethical approval of the study was given by the University of British Columbia/Children’s and Women’s Research Ethics Board (H12-00733) and (H16-02280). From each participant, written informed consent was obtained, and all procedures adhered to the ethical standards for human experimentation as outlined in the Helsinki Declaration of 1975 (revised in 2008).

At the time of recruitment, each participant received a Mini International Neuropsychiatric Interview (MINI) for the screening of DSM-IV Axis I Depression and the clinical diagnosis of major depressive disorder (MDD) and lifetime history of depression. [18]. At recruitment and the visits at 36 weeks gestation, maternal depressive symptoms were further assessed using the clinician-rated Hamilton Depression Rating Scale (HAM-D) [18]. The gestational age at 36 weeks ranged between 34.6 weeks and 37.14 weeks. Participants with a HAMD score ≥ 8 at the 36 weeks clinical visit were identified as having a symptomatic depressed mood as this has been clinically validated as a cut-off between symptomatic and asymptomatic depression [19]. After the exclusion of the participants treated with non-SRI antidepressants (n = 1), there were 82 placenta samples. Participants with a HAM-D < 8 and without the SRI treatment were selected as controls. Since depression alone can alter protein expression, there is indication bias in assessing the changes in protein expression associated with SRI exposure [20]. To properly control for the indication biases, we have analyzed the protein expression in subgroups of participants with similar indications separately. In this study, we focused on a dimensional approach to maternal depressive symptoms and not on whether mothers fulfilled a categorical clinical diagnosis of depression. With this approach, three groups of participants were identified to yield: 1) no or few depressive symptoms and no SRI treatment (control), 2) mild/moderate depressive symptoms without SRI treatment, and 3) mild/moderate depressive symptoms with SSRI treatment (Table 1).

**Table 1:** Participants information.

		Control	Depressive symptoms/ No-SRI treatment	Depressive symptoms/ SRI-treatment	Test statistics (p-value)
**n**		**18**	**34**	**18**	
**HAM-D at 36wk visit.** **mean (SD)**		4.5 (1.7)	10.7 (2.4)	13 (4.1)	p < 0.05
**SRI Type (n)**	Citalopram	–	–	7	
	Escitalopram	–	–	6	
	Fluoxetine	–	–	2	
	Sertraline	–	–	3	
**Length of SRI exposure** **(days, mean (SD))**				264.3 (26.1)	
**Delivery Mode (n)**	Cesarean	6	12	8	
	Vaginal	12	22	10	ns
**Fetal Sex**	Female: male	8: 10	15: 19	10: 8	ns
**Apgar Score at 5 min** (>7) **n (%)**		18 (100)	18 (100)	16 (89)	ns
**Maternal age at birth** **mean (SD)**		32.1 (3.1)	33.9 (3.1)	36.7 (3.9)	p < 0.05
**Gravidity** **median (IQR)**		0 (0.75)	0 (1)	0.5 (1)	ns
**Parity** **median (IQR)**		1 (0.75)	2 (1.75)	3 (1.75)	ns
**Gestational age at birth** **mean (SD)**		39.3 (1.7)	39.6 (1.3)	39.1 (1.3)	ns
**Birth weight (g)** **mean (SD)**		3346 (400)	3603 (394)	3336 (459)	<0.05
**Placental weight (g)** **mean (SD)**		564.5 (134.3)	622.1 (94.8)	589.4 (79.4)	ns
**Sample processing time (h)** **mean (range)**		40.5 (2-163)	61.0 (6-348)	43.2 (4.5-164)	ns

For continuous variables, normally distributed variables are presented as mean (SD = Standard Deviation) and the skewedly distributed variables as median (first, third quartile). Categorical variables are shown as total number (n). p-values and statistical significance associated with the test statistics (ANOVA), followed by a post hoc test with Tukey’s HSD between the groups, are reported (ns > p = 0.05). Abbreviations: SRI (serotonin reuptake inhibitor); HAM-D (Hamilton Rating Scale for Depression); g (grams); h (hours); IQR (Interquartile Ratio)

Participants exposed to SRIs were treated with either fluoxetine, paroxetine, sertraline, citalopram, or escitalopram for at least 75 days before delivery, and adherence to their treatment was assessed and recorded at each prenatal study visit. In addition, clinical information about the infants, such as gestational age at delivery, sex, birth weight, and Apgar score at 5 minutes, was collected. To minimize potential confounding, individuals with known medical or psychiatric conditions other than depression were excluded from the study. Exclusion criteria were maternal mental illness other than unipolar depression or anxiety, current substance abuse, gestational hypertension, diabetes, placental insufficiency, and any fetal anomaly such as congenital malformation and intrauterine growth restriction. Through clinician interviews and chart reviews, measures of obstetric history, prenatal medication use, and socio-demographic variables were obtained from all the participants.

### 2.2. Placenta sampling

Shortly after delivery, 1.5–2 cm^3^ sized chorionic villous tissue samples were collected from 4 different sites of the fetal side of the placenta; they were thoroughly washed with 1X PBS to eliminate any visible traces of blood and frozen for storage at –80°C until the analysis as previously described [21,22]. Tissues collected from all four sites were powdered using frozen mortar and pestle over the dry ice to prevent thawing and potential degradation [23]. For each sample,three replicates of 5 mg of powdered tissue samples were extracted using 7 M urea and 2 M thiourea in 100 mM ammonium bicarbonate buffer. The protein concentration of the resulting extract was measured using a Bradford assay [24]. Out of 82 samples extracted, 6 were excluded from subsequent protein analysis because of an inadequate number of proteins available (less than 100 µg of protein), leaving 76 samples to be further processed for the instrumental analysis.

For each sample, 100 µg of protein was diluted to 500 µl in 100 mM ammonium bicarbonate buffer, followed by reduction with dithiothreitol (DTT; 2 mM final) for 15 min, alkylated with iodoacetamide (3 mM final) for 30 min in the dark, before adding 2 µg of trypsin and incubating for 4 h at 37°C. Digests were then subjected to solid phase extraction using Oasis HLB cartridges (Waters, 1cc, 30 mg), with 100% methanol elution, dried down in a SpeedVac and reconstituted in 100 µl of 10% acetonitrile; 0.2% formic acid. Three pool samples were created by taking 10 µl of samples from each group to make the ion library for protein quantitation.

### 2.3. LC-MS/MS analysis

Samples were injected (20 μl) onto a Phenomenex Aeris PEPTIDE XB-C18 column at 40°C (100 × 2.1 mm, 1.7) using a Nexera UHPLC system (Shimadzu) with water (A) and acetonitrile (ACN) (B), both containing 0.1% formic acid, at a flow rate of 300 μL/min. The elution gradient started at 5% B for 2.5 min and was linearly increased to 30% B in 40 min, to 50% B in 2 min, then to 90% B in 2 min, held for another 2 min at 90% B. A TripleTOF 5600^+^ (quadrupole-time-of-flight) mass spectrometer (Sciex) equipped with a DuoSpray ion source in positive electrospray mode was used for high-resolution tandem mass spectrometry (HRMS/MS) analysis. Data-dependent analysis was performed on the pooled samples to collect HRMS/MS spectra and create an ion library of identified proteins [25]. The 76 samples were then analyzed individually with data-independent analysis (DIA or SWATH) with 100 variable precursor ion windows and a cycle time of 2.7 seconds. In both cases, the ion source parameters were set at 5 kV source voltage, 500°C source temperature, and 50 psi GS1/GS2 gas flows, with a declustering potential of 80 V [26]. The protein identification, quantification, and normalization were completed using OneOmics software from Sciex [27]. The protein identification, quantification, and normalization were completed using the cloud-based OneOmics Suite 3.1 from Sciex (26). DDA data from the pooled samples were searched with ProteinPilot 5.0.3 (Sciex) against the human UniProtKB/Swiss-Prot protein reference database with the following parameters: cysteine alkylation by iodoacetamide, trypsin enzyme, and global false discovery rate of 1% for proteins and peptides. The result file was used as the spectral ion library for SWATH quantitative data processing of the replicate sample sets. Up to 4 peptides per protein and 3 transitions per peptide were extracted and integrated using automatic retention time calibration, an XIC extraction width of 50 ppm, and a retention time window of 2 minutes [28]. Samples were normalized during data processing with most likely ratio (MLR) method. A total of 1471 proteins were extracted and subjected to differential analysis.

### 2.4. Determining covariates

The maternal and fetal characteristics and the sample degradation associated with sample processing time can have a crucial impact on the inferences about the placental protein expression [29,30]. Thus, we explored the relationship between maternal and fetal characteristics to identify any variables that may be associated with maternal depression status or SRI treatment. We used Pearson’s correlation test, Cramer’s V test, and Point-Biserial Correlation test to determine if any of the maternal/fetal variables and the sample processing time correlate to maternal depression status, HAM-D score, and their SRI treatment (S1 Fig). We further identified potential covariates that may affect the protein expressions in the placenta independently of depression or SRI treatment based on the literature and by confirming the association between the first five principal component scores (PC) of protein expression obtained through PCA analysis and the selected maternal/fetal characteristics and sample processing time (S1 Table) [30,31]. Education level, a commonly used proxy for socioeconomic status was found to be similar across groups. Self-reported smoking and alcohol use during pregnancy were also comparable among participants (i.e., absent all cases, except for one individual). Therefore, these variables were not included in the adjustment. It is shown that individuals’ ancestries impact protein biomarker levels [32]. However, in our study, most participants were of European ancestry, and no differences were noted between exposed, non-exposed, or depressed/not depressed groups as estimated using both self-reported and DNA methylation-based measures [33]. Therefore, the pregnant individuals’ ancestry was not selected as a covariate. Maternal BMI, which can potentially confound the placental proteomics profile, were unavailable for adjustment. The variables chosen as covariates were maternal age at birth, number of pregnancies, number of births, delivery mode, gestational age at birth, birth weight, Apgar score at 5 minutes, infant sex at birth, and sample processing time.

### 2.5. Data preprocessing and bioinformatics analysis

Amongst 76 samples with protein quantification, 6 samples were from the participants who had been treated with an SRI and were in remission (HAM-D < 8). These samples were excluded from the bioinformatics analysis (S2 Fig). After the exclusion, the bioinformatics analysis was conducted on 70 samples that were stratified into three groups: 1) no/fewer depressive symptoms and no SSRI treatment (control (n = 18)), 2) mild-moderate depressive symptoms without SSRI treatment (n = 34), and 3) mild-moderate depressive symptoms with SSRI treatment (n = 18) as mentioned in section 2.1. Preprocessing and the bioinformatics analysis of the proteomics data were completed using R (version 4.4.1). The data transformation, imputations, and differential expression analyses were conducted using the differential enrichment analysis of the proteomics data (DEP) package [34]. Protein quantities were transformed using the variance stabilization normalization (VSN) method to correct for systematic biases and ensure comparability between samples. Missing protein quantification values were handled by the minimum probability imputation method (q = 0.01). Missing values on the sample processing time were imputed using the missForest package, which implements the Random Forest algorithm. This approach produces a single imputed dataset and does not account for imputation uncertainty [35]. However, given the low proportion of missing data and the stability of our results in sensitivity analyses, the impact on our findings is minimal. Normalized Root Mean Squared Error (NRMSE) was close to 0 (0.02), indicating that the imputed values are close to the true values. The variables used as an input for the imputation were as follow: SRI exposure, HAMD/A score at 1st and 36 weeks visit, anxiety and depression status, gestational age at 1^st^ and 36 weeks visit, gestational age at birth, sample processing time, Edinburgh Postnatal Depression Scale (EPDS) score, maternal years of education, maternal alcohol use during pregnancy, maternal age birth, gravidity, parity, delivery type, season of birth, baby’s sex, baby’s weight, length and head circumference at birth, 5 minutes Apgar score, cord red blood cell count, as well as placental weight. The data reduction by principal component analysis (PCA) was made on the covariates (maternal age at birth, number of pregnancies(gravidity), number of births beyond 20 weeks (parity), delivery type, gestational age at birth, birth weight, Apgar score at 5 minutes, fetal sex and sample processing time) using the factoextra R package (S3 Fig) [36]. This allowed for the inclusion of primary sources of variation in the differential expression analysis model in the form of the first three principal components (PCs) while keeping the model less complex, preventing overfitting, and ensuring the statistical power of the differential expression analysis [37]. Then, using the DEP package, which uses the limma framework based on the empirical Bayes method [34], the protein expression profiles were compared between 1) the control group and mild/moderate depressive symptoms without SRI treatment group and 2) the mild-moderative depressive symptoms without SRI treatment and mild-moderate depressive symptoms with SRI treatment, through differential expression analysis. This allowed us to identify profiles of differentially expressed proteins and their log_2_ fold changes. The threshold for significance was set at FDR-adjusted p-value <0.05 with |log_2_ fold change| ≥ 0.5, with positive and negative signs indicating increasing and decreasing expression [38]. The over-representation analysis was conducted to identify which biological pathways are associated with these proteins. Three different over-representation analyses were completed using the Reactome and Kegg databases using the Reactome PA and clusterProfiler package [39,40]. The statistical parameters for the over-representation analysis were set for log_2_ FC > 1 and adjusted p.value <0.05. Multiple testing correction was performed using the Benjamini-Hochberg method to control for the false discovery rate (FDR) at 0.05 for both differential expression and pathway analyses.

## 3. Results

### 3.1. Participants information

Table 1 summarizes participants’ characteristics by study group. The maternal age at birth was significantly higher in the participants with depression and SRI treatment (p < 0.05) compared to the control group. According to ANOVA, there was a statistically significant difference in the overall birth weight among groups, but no significance was found after correcting for multiple comparison tests with Tukey’s HSD. As expected, the maternal depression scores significantly differed amongst all three groups (adj.p.val. < 0.05) based on ANOVA followed by Tukey’s HSD Post hoc test. The Point-Biserial Correlation test and Pearson’s correlation tests showed that maternal age is positively associated with depression, HAM-D score at 36 weeks, and SRI exposure (**S1 Fig**). Due to the small sample size per drug among the 18 individuals exposed to SRIs, statistically meaningful differential protein expression analysis by SRI type was not feasible. Principal Component Analysis (PCA) of protein expression data indicated that SRI drug type did not drive the major sources of variance, with PC1 and PC2 accounting for 13.36% and 9.98% of the total variance, respectively (S4 Fig).

### 3.2. Proteins differential expression and pathway analysis

**Fig 1:**
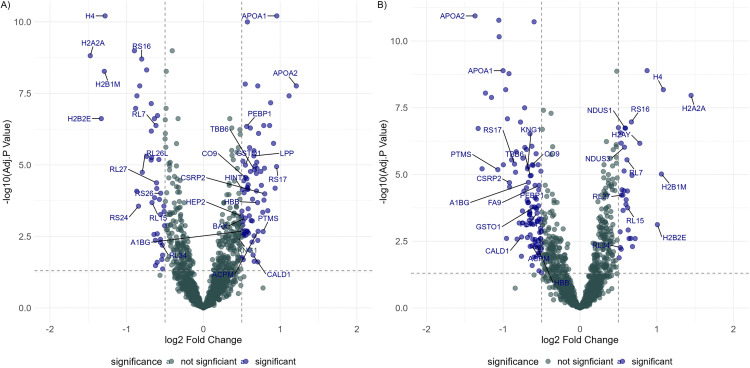
The expression of senescence-related proteins differentially expressed in the group with mild-moderate depressive symptoms without SRIs is subdued in the mild-moderate depressive symptoms with SRI treatment group. **(A)** Placental protein expression from depressed women without SRI treatment was compared to the non-depressed controls. **(B)** The expression of proteins in the placenta from mild-moderately depressed individuals with SRI treatment was compared to the individuals with mid-moderate depression without SRI treatment. The x-axis represents the log_2_ fold change in protein expression, while the y-axis represents the -log10 adjusted p-value. Proteins with a significant fold change (|log_2_ FC| ≥ 0.5) and adjusted p-values (adj.p < 0.05) are colored blue, while non-significant proteins are colored green. Dashed lines indicate thresholds for significance (adj.p < 0.05 and |log_2_FC| ≥ 0.5). Proteins with increased expression (positive log_2_FC) are shown in the top-right quadrant, while those with decreased expression (negative log_2_FC) are in the top-left quadrant.

From the 70 placentas (chorionic villi) analyzed, 1471 proteins were identified and subjected to differential expression analysis. In the individuals with mild-moderate depressive symptoms without SRI treatment, 117 proteins were differentially expressed, of which 75 proteins were significantly increased, and 47 proteins were significantly decreased compared to the control group (|log_2_ FC| ≥ 0.5, adj.p < 0.05). Compared to individuals with mild-moderate depressive symptoms without SRI treatment, 136 proteins were differentially expressed in the placenta from the individuals with mid-moderate depressive symptoms treated with SRIs, of which the expressions of 34 proteins were increased. In comparison, 105 proteins were decreased (|log_2_ FC| ≥ 0.5, adj.p < 0.05) (Fig 1A and B).

In the placentas of mild-moderately depressed individuals without SRI treatment, the proteins with the most decreased expression were histone proteins H2A2A, H2B2E, H2B1M, and H4 (|log_2_FC| > 1, adj.p < 0.05). In contrast, the proteins that increased the most in expression were Thymosinß-4 (TYB4), apolipoproteins such as APOA1 and APOA2, and ribosomal protein RS17 (|log_2_FC| > 1 adj.p < 0.05). In the case of placental tissues from individuals who have mild-moderate depressive symptoms treated with SRIs compared to those without SRI treatment, histone proteins were significantly elevated. In contrast, thymosinß-4 (TYB4), apolipoproteins, synuclein alpha (SYUA), and alpha hemoglobin stabilizing protein (AHSP) proteins were significantly reduced (|log_2_FC| > 1, adj.p < 0.05).

**Fig 2:**
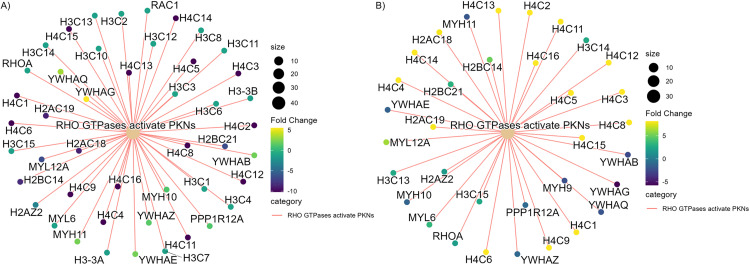
RHO GTPases that activate the PKN Reactome pathway are over-represented in (A) depressed without SRI and (B) depressed with SRI groups. In the placentas of depressed individuals without SRI, compared to healthy controls, the pathways associated with RhoGTPase activation (GeneRatio = 46/492, p.adj = 1.81E^-34^) are overrepresented with many of the related proteins, except for the Histone subunits, with positive fold change (node color gradient green ~ yellow). This pathway was also over-represented in depressed with SRIs compared to depressed without SRIs (GeneRatio = 33/512, p.adj = 9.85E^-19^), with associated proteins negative fold changes (node color gradient blue). Each cluster and the beige node represent the Reactome pathway, and the smaller nodes represent differentially expressed proteins with color intensity representing the direction and magnitude of fold change (positive fold change in yellow, negative fold change in blue). The node size represented the number of proteins expressed in the pathway.

Compared to the control, the individuals with mild-moderate depression without SRI showed decreased expression in the nucleosome histone proteins (H2A, H2B, H3, H4), the 40S and 60S ribosomal proteins, and the components of electron transport chain such as Complex I (NADH oxidoreductase), components such as NDUS (NADH dehydrogenase (Ubiquinone) Fe-S protein 6), NDUA (NADH Dehydrogenase [Ubiquinone] 1 Alpha Subcomplex) and complex IV (cytochrome c oxidase). Furthermore, the antioxidant enzymes such as GLRX1 and 3 (glutaredoxin 1 and 3) and PRDX 6 (peroxiredoxin 6) (|log_2_FC| > 0.3, adj.p < 0.05), and cell structural proteins such as TUA1A (tubulin α 1a), TBB6 (tubulin ß 6 class V) (|log_2_FC| > 0.4, adj.p < 0.05) were elevated in the placentas from depressed individuals without SRIs compared to healthy controls. The alterations in proteins related to cell morphology are further supported by the over-represented Reactome pathway “RHO GTPases activate PKN” in both the depressive symptoms without SRI and the depressive symptoms with SRI groups with opposite directions in its expression. The expressions of Rho GTPase activity proteins, such as the YWHA family of proteins, RAC1 and Myosin, were increased in the group with depressive symptoms without SRI treatment (GeneRatio = 46/492, adj.p = 1.81E^-34^) (Fig 2A). In contrast, the expressions in the SRI-treated group decreased (GeneRatio = 33/512, adj.p = 9.85^E-19^) (Fig 2B).

In the placentas of mild-moderately depressed individuals without SRI treatment compared to the controls, the hallmarks of senescence such as the loss of histone protein expression, diminished ribosomal proteins, increase in oxidative phosphorylation (OXPHOS) related proteins and increased release of senescence-associated secretory phenotype (SASP) proteins were identified [41]. On the contrary, in depressed individuals treated with SRIs, the histone protein, and oxidoreductase proteins were significantly increased, and antioxidant enzymes and cell structural proteins were reduced compared to the mild-moderately depressed individuals without the SRI treatment. Furthermore, there were fewer elevated SASPs observed in the individuals who have mild-moderate depressive symptoms under SRI treatment than in the mild-moderately depressed individuals without SRI treatment. From the SASP Atlas, we obtained a more detailed list of SASPs amongst proteins that were significantly differentially expressed in the placentas of individuals with mild-moderate depressive symptoms treated with and without SRI treatments [42]. Out of 122 differentially expressed proteins (|log_2_FC| ≥ 0.5, adj.p < 0.05) in the mild-moderately depressed individuals without SRI, the expression of 13 SASPs was elevated relative to the controls. These proteins included cell structure and cytoskeleton dynamic proteins such as CALD1 (caldesmon1) and CSRP2 (cysteine and glycine-rich protein 2) and oxidative stress response proteins such as GSTO1(glutathione S-transferase omega-1). On the other hand, there were 19 decreased SASP proteins in the placentas from individuals with mild-moderately depressive symptoms with SRI treatment compared to those without SRI treatment, with a significant decrease in the proteins involved in cytoskeletal dynamics. These proteins included CALD1, CSRP2, PALLD (palladin), CNN3 (calponin3) and CRIP2 (cysteine-rich protein 2) (**Table 2**).

**Table 2:** Differentially expressed proteins identified as SASP according to SASP Atlas. (A) Placentas of individuals with mild-moderate depressive symptoms without SRIs compared to the control and (B) Placentas from the individuals with mild-moderate depressive symptoms with SRIs compared to the individuals with mild-moderate depressive symptoms without SRI treatment.

A) SASPs differentially expressed in depression without SRI vs healthy controls						B) SASPs differentially expressed in SRI exposed vs depressed without SRI					
Gene name	Name	log_2_FC	AveExpr	adj.P.Val	Expression	Gene name	Name	Log_2_FC	AveExpr	adj.P.Val	Expression
PTMS	Parathymosin	0.78	13.40	2.15E^-03^	↑	CD59	CD59 molecule	0.59	14.69	1.84E-07	↑
PEBP1	Phosphatidylethanolamine binding protein 1	0.56	15.36	4.53E^-07^	↑	ECH1	Enoyl-CoA hydratase 1	0.55	15.87	3.74E-05	↑
LPP	LIM domain containing preferred translocation partner in lipoma	0.66	11.22	4.84E^-06^	↑	CALD1	Caldesmon 1	−0.76	14.31	2.17E-03	↓
HBB	Hemoglobin subunit beta	0.71	22.09	2.08E^-04^	↑	A1BG	Alpha-1-B glycoprotein	−0.67	15.30	1.94E-05	↓
CSRP2	Cysteine and glycine rich protein 2	0.80	12.64	1.03E^-04^	↑	APOA1	Apolipoprotein A1	−1.00	18.69	1.28E-09	↓
COTL1	Coactosin like F-actin binding protein 1	0.66	14.42	3.19E^-06^	↑	APOC3	Apolipoprotein C3	−0.79	14.62	6.50E-04	↓
A1BG	Alpha-1-B glycoprotein	0.51	15.39	2.06E^-03^	↑	CNN3	Calponin 3	−0.55	14.19	4.23E-06	↓
APOA1	Apolipoprotein A1	0.96	18.75	6.19E^-11^	↑	COTL1	Coactosin like F-actin binding protein 1	−0.64	14.33	1.10E-05	↓
APOC3	Apolipoprotein C3	0.77	14.62	4.54E^-04^	↑	CRIP2	Cysteine rich protein 2	−0.54	12.20	3.19E-02	↓
BAX	BCL2 associated X, apoptosis regulator	0.54	10.78	7.22E^-04^	↑	CSRP1	Cysteine and glycine rich protein 1	−0.51	13.51	6.11E-04	↓
CALD1	Caldesmon 1	0.65	14.12	1.41E^-02^	↑	CSRP2	Cysteine and glycine rich protein 2	−0.94	12.69	1.01E-05	↓
GSTO1	Glutathione S-transferase omega 1	0.55	15.46	1.01E^-05^	↑	GARS	Glycyl-tRNA synthetase 1	−0.52	10.94	2.17E-03	↓
HINT1	Histidine triad nucleotide binding protein 1	0.51	13.68	7.32E^-05^	↑	GSTO1	Glutathione S-transferase omega 1	−0.61	15.45	2.02E-04	↓
FAM3C	FAM3 metabolism regulating signaling molecule C	−0.83	11.62	1.73E^-08^	↓	HBB	Hemoglobin subunit beta	−0.57	22.08	4.21E-03	↓
ANXA1	Annexin A1	−0.50	18.17	2.65E^-07^	↓	HTRA1	HtrA serine peptidase 1	−0.51	12.82	6.28E-03	↓
CKAP4	Cytoskeleton associated protein 4	−0.57	13.98	1.61E^-04^	↓	PALLD	Palladin, cytoskeletal associated protein	−0.63	11.29	6.50E-04	↓
LAMP1	Lysosomal associated membrane protein 1	−0.51	11.58	4.25E^-04^	↓	PEBP1	Phosphatidylethanolamine binding protein 1	−0.54	15.32	3.74E-05	↓
LAMP2	Lysosomal associated membrane protein 2	−0.50	13.44	1.88E^-04^	↓	PTMS	Parathymosin	−1.08	13.50	6.34E-06	↓
LMNA	Lamin A/C	−0.50	17.03	1.23E^-06^	↓	PZP	Pregnancy Zone Protein	−0.66	10.61	6.73E-04	↓
						SCRN1	Secernin 1	−0.66	11.63	2.96E-02	↓
						UGDH	UDP-glucose 6-dehydrogenase	−0.58	12.71	7.40E-05	↓

### 3.3. Mild-moderate depressive symptoms without SRI treatment increase the expression of proteins linked to amyloid fiber formation

**Fig 3:**
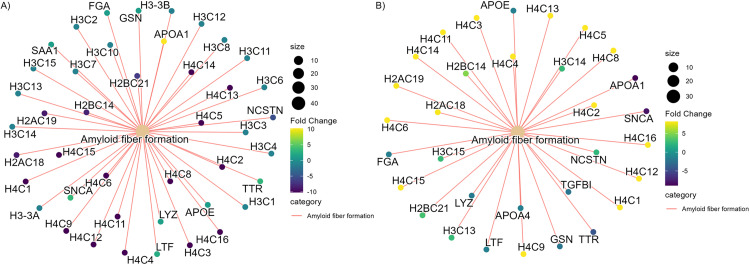
Amyloid fiber formation pathways are over-represented in (A) depressed with SRI and (B) depressed without SRI groups with opposite protein fold changes. According to Reactome Pathway, Amyloid fiber formation is over-represented in mild-moderately depressed individuals without SRI (compared to controls) (GeneRatio = 43/492, adj.p = 1.87E^-^^27^) and mild-moderately depressed with SRI (compared to mild-moderately depressed without SRI) (GeneRatio = 32/512, adj.p = 6.40E^-16^). The expression of proteins represented in the Amyloid fiber formation pathway was elevated in the placentas of the individuals with depressive symptoms without SRIs. In contrast, the expression of these proteins decreased in the mild-moderately depressed individuals with SRI treatment. Each cluster and the beige node represent the Reactome pathway, and the smaller nodes represent differentially expressed proteins with color intensity representing the direction and magnitude of fold change (positive fold change yellow, negative fold change). The node size represented the number of proteins in the pathway.

In the placentas of mild-moderate depressed individuals without SRI treatment compared to the controls, the major players of amyloid formation such as APOA1 (apolipoprotein-1), SNCA (alpha-synuclein) and TTR (transthyretin) were increased while NCSTN (nicastrin) a crucial component of γ-secretase complex responsible for cleaving the Amyloid Precursor Protein (APP) was decreased (|log_2_FC| > 0.5, adj.p < 0.05). The pathway analysis by Reactome (GeneRatio = 43/492, adj.p = 1.87E^-27^) showed the over-representation of the amyloid formation-related pathway (Fig 3A). KEGG pathway analysis further supported the association of amyloid formation pathway in the placentas from the individuals who have mild-moderate depressive symptoms with without SRI treatment, by suggesting an increase in the proteins related to prion disease, a disease caused by the subclass of amyloid (Fold Enrichment = 2.71, GeneRatio = 35/412, adj.p. = 2.29E^-6^) (S5 Fig). The proteins associated with amyloid fiber formation were also over-represented in the placentas from the mild-moderately depressed individuals with SRI treatment, but the expressions of these amyloid-associated proteins were diminished (GeneRatio = 32/512, adj.p = 6.40E^-16^) (Fig 3B). In addition, pregnancy zone protein (PZP), an inhibitor of protein misfolding, which was not differentially expressed in the individuals with depressive symptoms without SRI treatment compared to the control group, was significantly reduced in the mild-moderately depressed individuals treated with SRI (log_2_FC = −0.66, adj.p < 0.05)).

### 3.4. Mild-moderate antenatal depression without SRI treatment is associated with platelet degranulation and coagulation cascade, and the SRI treatment attenuates the effect

**Fig 4:**
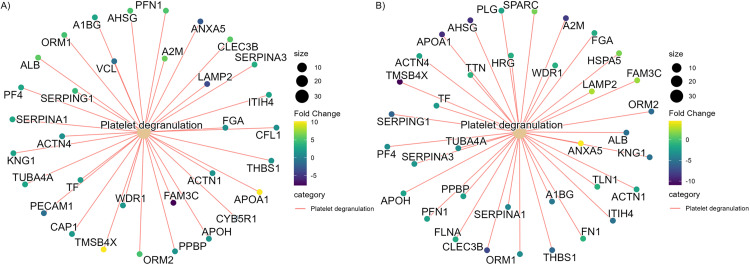
Platelet degranulation pathways are over-represented in both (A) the individuals with mild-moderate depressive symptoms without SRI relative to control and (B) the individuals with mild-moderate depressive symptoms with SRI treatment relative to mild-moderately depressed individuals without SRI treatment. Platelet degranulation pathway is over-represented in the mild-moderately depressed group without SRI treatment compared to control (geneRatio = 33/492, adj.p = 1.24E^-15^) with positive fold change in the majority of represented proteins (fold change gradient = green-yellow). In the individuals with mild-moderate depressive symptoms with SRI treatment compared to those with mild-moderately depressive symptoms without SRI, the expression of the proteins involved in platelet degranulation pathways (GeneRatio = 36/512, p.adj = 4.75E^-17^) was significantly reduced (fold change gradient = dark green blue. Each cluster and the beige node represent the Reactome pathway, and the smaller nodes represent differentially expressed proteins with color intensity representing the direction and magnitude of fold change. Proteins with more considerable negative fold changes have a darker blue color, while the positive fold changes are presented with lighter green to yellow.

In the placentas of individuals with mild-moderate depressive symptoms without SRI treatment compared to the control group, the expression of procoagulant proteins were increased, such as kininogen 1(KNG1), platelet factor 4 (PF4), which are released during platelet degranulation. The expression of coagulation factor IX (F9), heparin cofactor II (HEP2), and complement component 9 (CO9) secreted during the coagulation cascade were also enhanced (log_2_FC>0.45, p.adj. < 0.05 (Fig 1A). On the contrary, the expression of these proteins was significantly decreased in mild-moderately depressed individuals treated with SRI (log_2_FC < 0.6) (Fig 1B). The pathway analysis based on the Reactome database also suggests the over-representation of the platelet degranulation pathway in control and the individuals with depressive symptoms and SRI treatment, with most of the proteins involved in the pathway having positive fold changes in the depressive symptoms without SRI group (geneRatio = 33/492, adj.p = 1.24E^-15^), and negative fold change in the depressive symptoms with SRI group (gene ratio = 36/512, adj.p = 4.75E^-17^). The proteins such as Thymosin ß-4 (TMSB4X; also known as TYB4), α-2-Macroglobulin(A2M), APOA1 and α-2- S-glycoprotein (AHSG; also known as FETUA) had one of the largest negative fold changes amongst the proteins represented in the pathway (Fig 4A and B).

## 4. Discussion

In the present study, we showed that placental tissues delivered from the pregnant individuals with mild to moderate depressive symptoms without SRI treatment exhibited altered expression of proteins involved in biological processes related to cellular senescence, platelet degranulation, and amyloid formation relative to non-depressed controls. This reflects pathways known to be associated with placental pathology and adverse pregnancy outcomes [43–45]. These proteomic signatures found in the placentas of mild to moderately depressed individuals without SRIs were reversed in the placentas of individuals with mild to moderate depressive symptoms treated with SRIs.

Senescence refers to the aging of the cells with a permanent halt in cellular division without dying. The accumulation of old cells that remain metabolically active releases substances that may damage nearby healthy cells, leading to pathology [46].Shortening of telomeres and progressive DNA damage by oxidative stress are the initiating factors of senescence that lead to the arrest of the cell cycle [47]. The cells in senescence are presented with various features such as shortened telomeres, inflammation, oxidative stress, and reorganization of the microtubule cytoskeleton, and during this process of senescence, cells release soluble proteins called SASP [41].

According to the “SASP Atlas”, the proteomic database which provides SASP factors originating from multiple senescence inducers and cell types, the placental tissue from individuals with mild-moderate depressive symptoms without the SRI treatment in the present study exhibited altered expression of various SASP factors, suggesting potential placental senescence associated to maternal depressive symptoms. Additionally, in the placentas of mild-moderately depressed individuals without SRI treatment, reduced expression of histone proteins and increased expression of Peroxiredoxins (Prxs) and Glutaredoxins (Grxs) was observed, which is indicative of oxidative stress, and deterioration of OXPHOS. There are few studies focused on the placenta compared to other tissues. Oxidative stress has been suggested to induce senescence in the placenta. Indeed, an increased nuclear localization of p21 and phosphorylated histone γH2AX proteins expression was shown in syncytiotrophoblast of placental explants under the oxidative stress induced by reoxygenation and hydrogen peroxide [48]. Furthermore, a recent study demonstrated a post-translational modification of histones in placental senescence and preeclampsia [49]. Reduced expression of histone proteins, such as that observed in placentas from cases of untreated depression, is a hallmark feature of cellular senescence in other cell types. For example, in fibroblast cells, reduced expression of H3 and H4 resulted in chromatin condensation during replicative and stress-induced premature senescence, while H2A.Z, which regulates CDKi, was downregulated during oncogene-induced senescence [50–52]. Reorganization of the microtubule cytoskeleton is one of the features of cellular senescence [53,54]. Here, the placentas of individuals who have mild-moderate depressive symptoms without SRI treatment, relative to individuals with no or fewer depressive symptoms, had increased expression of microtubule cytoskeleton components such as tubulin isoforms, and the cell adhesion and migration molecules such as CSRP proteins and ACTN. In addition, the increase of 14-3-3 (YWHA family) proteins and the decrease of RhoGTPase proteins, such as RHOA and Rac1, that directly and indirectly interact to regulate the RHOA/ROCK and Rac1/PAK pathways, suggests the hindrance of cellular contractility and the movement [55]. The RhoA/ROCK pathway changes induce cell stiffness and nuclear blebbing, leading to cytoskeletal reorganization. Cytoskeletal reorganization is one of the characteristics of senescence. Dysfunctional actin filaments enhanced by DNA damage and oxidative stress cause uncontrolled cell proliferation and tumorigenesis, leading to cellular senescence [56,57]. Recent studies have shown that maternal depression could accelerate placental aging through DNA methylation and telomere shortening [58,59]. While studies focusing on the placental release of SASP associated with maternal depression are unavailable, reduced antioxidant capacity, mitochondrial dysfunction, elevation of proinflammatory markers, and the changes in cytoskeletal rearrangements associated with Rho proteins and the downstream pathways have been identified in the serum, plasma and the brain of depressed general population, prenatal and postpartum women [54,60–62].

The senescence-related proteins that are significantly altered in the depressed individuals without SRIs compared to the healthy control group showed opposite expression patterns in those who received SRI treatment, indicating an association between SRI exposure and placental protein expression that warrants further investigation. Studies that explain the molecular pathway involved in SRIs’ action on cellular senescence have yet to be done. However, several studies in non-placental tissues have shown that SRIs reduce the markers of oxidative stress and inflammation [63,64]. Furthermore, Rampersaud et al., demonstrated that shorter telomere length is associated with poor antidepressant response in depressed individuals, suggesting that SRI treatment could potentially be related to cellular senescence [65]. Nonetheless, there’s also contradicting evidence suggesting that SRI treatment induces cell inflammation and senescence through elevation of p53 MAPK activation in fetal membrane treated with fluoxetine [66]. Placental senescence is a natural and necessary process of placental development. The senescence of trophoblast is a physiological phenomenon that naturally occurs with the progress of pregnancy. During the syncytialization of cytotrophoblast to syncytiotrophoblast, cell fusion-induced senescence occurs through the activation of ERVWE-1, and the senescence supports the viability of syncytium through its resistance to apoptosis [67]. Delayed placental senescence is associated with IUGR pathology in humans and abnormal placental morphologies in rodent placentas [41]. On the other hand, accelerated aging of the placenta accompanied by the release of SASP, shortened telomere, ROS, and inflammation in the placenta has also been linked to placental stress, IUGR, and stillbirth, suggesting that both the delay and the premature aging of the trophoblast can result in average pregnancy outcome. [68]. Our results showed changes in the expression of proteins, which are the markers of senescence. However, to confirm the placental senescence linked to depressive symptoms during pregnancy and SRI treatment, additional assays to identify primary markers of senescence, such as p16/Rb network and SA-ß-gal, which are the markers of cell cycle arrest and structural changes, respectively, should be carried out [69].

The placental tissue from the individuals with mild-moderate depressive symptoms without SRI treatment showed increased expression of proteins related to platelet activation and degranulation compared to the individuals with no or fewer depressive symptoms. The link between depression and enhanced platelet activation in depressed patients has been shown. Indeed, an increased responsivity to the platelet-activating stimuli (i.e., collagen, thrombin), the increased plasma level of platelet granule contents indicative of platelet activation and subsequent coagulation process (i.e., β-thromboglobulin, factor four (PF4 factors VII and X)), and the platelet aggregation have been shown in patients with the major depressive disorder as compared to healthy controls [70,71]. The platelet-derived proteins in placental tissues play an important role in uterine vascular remodeling, differentiation, and cytotrophoblast invasion [72,73]. To our knowledge, this is the first study to show that mild-moderate depressive symptoms during pregnancy elevates the proteins associated with platelet degranulation in the placental tissue.

The activation and degranulation of platelets could drive amyloid formation. The accumulation of these fibrous proteins is associated with various pathologies [74]. In their alpha granules, platelets contain high amyloid precursor proteins (APP) and amyloid beta (Aß). Upon platelet activation by hemostatic agonists such as Thrombin, the platelet release Aß1-40 and pathogenic Aß1-42 along with coagulation factors and adhesion molecules, contributing to the thrombosis at the site of platelet adhesion [75,76]. We have identified a significant increase of SNCA, TTR, APOA1, and light chain variable of immunoglobulin protein, the proteins whose aggregation leads to different types of amyloidosis [77]. Moreover, α-2-macroglobulin(α2M), the inhibitor of protein aggregation, and Nicastrin (NICA), a crucial component of the γ-secretase complex responsible for cleaving the Amyloid Precursor Protein (APP), were reduced in the placental tissues from depressed mothers compared to the healthy control in our study [78,79]. The association of amyloidosis and depression has been explored mainly in late-life major depression accompanied by cognitive decline and neurological diseases [80–82]. One possible explanation for the link between depression and amyloidosis may be through the serotonin system. 5-HT_2A_, 5HT_2B,_ and 5-HT_3_ receptor antagonists and serotonin transporter inhibitors have been shown to decrease the amyloid formation in the hippocampus, cortical neurons, and cerebrospinal fluid [76,83–85]. The effect of SRIs on amyloid formation has been demonstrated clinically and in vivo. Brain imaging of cognitively normal patients in late life without SRI treatment had higher cortical Aß than those treated with SRI [76]. Furthermore, the in-vivo study has shown that Citalopram reduces the formation of new amyloid plaques and blocks the growth of existing ones [85]. According to Cirrito et al., serotonin reduces amyloid production through the activation of the serotonin receptor-mediated ERK pathway, which increases in α and γ-secretase cleavage activity, and the rapid activation of ERK pathways and the phosphorylation of these secretase enzymes following the SRI treatment allows fast amyloid reduction response [76,85,86]. Thus, our result suggests that a change in the placental serotonin system could be associated with depression, affecting the expression of proteins that mitigate the amyloid formation in the placentas of depressed mothers without SRI treatment.

To this date, the potential link between antenatal depression and placental amyloidosis has never been explored, and the identification of amyloid-related proteins differentially expressed in the placenta provides limited information to establish a clear association. Identifying and quantifying actual plaque and the amyloid proteins in the placenta should be completed to confirm the placental amyloid formation linked to antenatal depression.

The elevated expression of proteins associated with platelet activation, coagulation, and amyloid formation in the placentas from mild-moderately depressed individuals without SRI treatment observed in our study may entail the interference of placental hemostasis [87,88]. Maintenance of hemostatic balance at the maternal-fetal interface is one of the key factors that allows the placenta to carry out its primary function of exchanging oxygen, nutrients, and waste between the mother and the fetus. The formation of perivillous fibrin-type fibrinoids at the surface of placental villi through activation of platelet is a normal process as the continuous cycle of deposition and breakdown of fibrinoids at the villous surface is a key factor in regulating the intervillous hemodynamics, shaping of villous tree and intervillous space. When there is an abnormal branching of the villous, these newly formed sprouts are degenerated by the local stasis induced by the maternal coagulation at the site [89,90]. However, the excessive placental thrombosis and the over-formation of fibrin lead to abnormal remodeling of the placental bed early in the pregnancy. This can lead to pre-eclampsia and placental vascular lesions, resulting in neonatal complications such as fetal thrombotic vasculopathy [91,92].While remaining speculative without the histopathological examination of the placenta, these findings suggest the possibility of untreated antenatal depression impairing the placental hemostasis, potentially contributing to poor placenta development and adverse fetal outcomes.

While our study provides valuable insights, it has some limitations that should be considered. Participants were grouped according to symptom severity assessed using the HAM-D, and the individuals with scores near the cut-off for depression versus non-depression may not differ in a clinically meaningful manner. This could have introduced misclassification bias. Another limitation of our study is the small sample size. Due to the small number of participants exposed to different types of SRIs, statistically meaningful differential expression analysis of the proteomes by drug type was not feasible. Another limitation is the lack of data on the plasma drug levels which could have provided additional insight to exposure and biological effects. The proteomic data alone do not directly reflect the functional effect of depression and SRI treatment on the placenta. Despite these limitations, our findings provide a foundation for further investigation with larger cohorts and integration of functional assays, such as assessment of senescence markers like telomere length or senescence-associated β-galactosidase activity (SA-β-gal), could build on our results to better understand the biological mechanisms involved. Furthermore, the measurement of amyloid plaques and placental hemodynamics could further validate the effect of antenatal depression and SRI treatment on placenta hemostasis and subsequent vasculopathy. In summary, these results suggest that antenatal depression and SRI treatment are associated with changes in placental protein markers linked to senescence and hemostasis, which may potentially affect placental development and function. Even though further research is needed to explore these effects in depth, this work provides data supporting the need for more comprehensive investigations into how maternal mental health and pharmacological treatment affect placental biology and fetal outcomes.

## Supporting information

S1 Table Association between the first five Principal Components (PC) scores obtained from Principal Component Analysis (PCA) on the protein expression and potential covariates (delivery mode, baby’s sex, Apgar score, maternal age, gravidity, parity, gestational age, birth weight and sample processing time). R^2^ (coefficient of determination) and P-value (statistical significance) obtained from regression analysis show how each variable explains variations in the Principal Component Scores of protein expression.
(DOCX)

S1 Fig Relationship between maternal/fetal variables a) Cramer’s V for the association between categorical variables V) Pearson’s correlation test between continuous variables and C) Point-Biserial correlation between continuous and categorical variables. Color gradient shows the degree of association. Cramer’s V is a measure of effect size, derived from the chi-square statistic. Values range from 0(no association) to 1 (perfect association). Statistical significance of the correlation is provided for the Pearson’s and Point-Biserial correlation. *H*_*o*_ states that there is no relationship between the two variables (r = 0). P < 0.05 suggests that the observed is unlikely due to the null hypothesis, leading to the rejection of *H*_*o*_. 1 indicates a positive relationship and −1 indicates a negative relationship.
(TIF)

S2 Fig After excluding participants prescribed non-SRI antidepressants, samples with low protein concentration, and participants with HAMD<8 with SRI treatment, 70 samples were subjected to bioinformatics analysis. (TIF)

S3 Fig Scree plot showing the proportion of variance explained by each principal component from the PCA of covariates (maternal age at birth, number of pregnancies, number of births, delivery type, gestation age at birth, birth weight, Apgar score at 5 minutes, fetal sex, and sample processing time). The x-axis shows the dimension, and the y-axis shows the percent of explained variance.
(TIF)

S4 Fig Principal component analysis of protein expressions amongst 18 individuals with depressive symptoms and SRI treatment suggests that the drug type does not drive the major source of variance of protein expression. The X-axis and Y-axis show the percentage of variance explained by Component 1 and 2, respectively. The color of the dots indicate different SRIs (pink:Citalopram, green: Escitalopram, blue: Fluoxetine, purple: Sertraline).
(TIF)

S5 Fig Prion disease pathway is over-represented in depressed mothers without SSRI treatment compared to healthy control according to the KEGG pathway. The green and red colors indicate the proteins within the pathway with decreased and increased expression, respectively.
(TIF)
