# Supplementary material for: Effect of depression and serotonin reuptake inhibitors antidepressant treatment during pregnancy on protein expression in the human placenta: A quantitative proteomics analysis
Source: PLoS One. 2025 Dec 11;20(12):e0322090. doi: 10.1371/journal.pone.0322090 (PMC12698013; doi:10.1371/journal.pone.0322090)
Supplement: S1 Table — R2 (coefficient of determination) and P-value (statistical significance) obtained from regression analysis show how each variable explains variations in the Principal Component Scores of protein expression. (DOCX) [file pone.0322090.s001.docx]

|  | PC1 | | PC2 | | PC3 | | PC4 | | PC5 | |
| --- | --- | --- | --- | --- | --- | --- | --- | --- | --- | --- |
|  | R^2^ | p-value | R^2^ | p-value | R^2^ | p-value | R^2^ | p-value | R^2^ | p-value |
| Delivery mode | 3.22E-06 | 9.88E-01 | 5.85E-03 | 5.29E-01 | 2.94E-02 | 1.56E-01 | 4.24E-04 | 8.66E-01 | 3.66E-02 | 1.13E-01 |
| Baby’s sex | 1.25E-02 | 3.58E-01 | 1.99E-02 | 2.44E-01 | 1.78E-02 | 2.71E-01 | 1.10E-03 | 7.85E-01 | 2.46E-02 | 1.95E-01 |
| Apgar score | 3.41E-02 | 1.26E-01 | 6.22E-03 | 5.16E-01 | 3.87E-04 | 8.72E-01 | 1.07E-03 | 7.88E-01 | 1.98E-02 | 2.46E-01 |
| Maternal age | 5.54E-02 | 4.98E-02* | 1.51E-03 | 7.49E-01 | 7.58E-02 | 2.11E-02* | 2.51E-02 | 1.91E-01 | 4.13E-05 | 9.58E-01 |
| Gravidity | 1.54E-03 | 7.47E-01 | 7.24E-03 | 4.84E-01 | 9.93E-03 | 4.12E-01 | 2.71E-03 | 6.69E-01 | 1.34E-02 | 3.40E-01 |
| Parity | 6.20E-05 | 9.48E-01 | 4.68E-03 | 5.74E-01 | 9.32E-03 | 4.27E-01 | 6.08E-04 | 8.39E-01 | 7.35E-03 | 4.80E-01 |
| Gestational age | 6.79E-02 | 2.94E-02* | 1.53E-02 | 3.08E-01 | 5.15E-02 | 5.88E-02 | 4.68E-04 | 8.59E-01 | 1.64E-02 | 2.91E-01 |
| Birth weight | 2.69E-02 | 1.75E-01 | 5.66E-02 | 4.73E-02* | 2.23E-02 | 2.17E-01 | 9.84E-03 | 4.14E-01 | 3.02E-04 | 8.86E-01 |
| Sample processing time | 1.47E-02 | 3.17E-01 | 7.39E-02 | 2.28E-02* | 1.45E-03 | 7.54E-01 | 2.62E-01 | 5.92E-06* | 1.22E-01 | 2.98E-03* |
